# Redefining Alzheimer’s diagnosis: the role of Lumipulse in bridging the gap

**DOI:** 10.1097/MS9.0000000000004447

**Published:** 2025-11-25

**Authors:** Maria Mohsin, Tayyaba Ashraf, Hermann Yokolo

**Affiliations:** a Department of Internal Medicine, Dow University of Health Sciences, Karachi, Pakistan; b Department of Research, Medical Research Circle (MedReC), Goma, DR Congo

## Introduction

Alzheimer’s disease (AD) remains the leading cause of dementia, accounting for 60–80% of cases^[1]^. In 2020, more than 55 million people worldwide were living with some form of dementia, and this number could reach 139 million by 2050^[2]^. This rapid increase reflects the urgent need for earlier and more equitable detection. Despite advances, diagnosis is still too often delayed, made only after cognitive symptoms are well established. AD is a neurodegenerative disease characterized by the accumulation of abnormal proteins, notably hyperphosphorylated Tau and beta-amyloid, which disrupt neuronal communication and lead to the death of brain cells^[1,3]^. The majority of cases are sporadic and occur after age 65, often associated with mutations in the APOE gene^[4]^. In its rarer, early-onset form, mutations in the APP, PSEN1, and PSEN2 genes are involved^[1]^. Early diagnosis is essential to initiate disease-modifying treatments and plan for care, but detection before the onset of symptoms remains challenging. PET imaging and cerebrospinal fluid (CSF) biomarkers remain accurate but are expensive or invasive, while lumbar puncture remains a major barrier to patient adherence^[3]^. Hence the need for more accessible and less invasive tools. This article aims to critically examine the evolution of AD diagnosis in light of the Food and Drug Administration (FDA)’s approval of the Lumipulse G pTau217/β-amyloid 1-42 blood test, the first of its kind. It seeks to highlight the potential of this test to transform clinical practice by making screening earlier, more accessible, and more equitable, while also discussing its methodological limitations and the ethical and organizational challenges associated with its integration into healthcare systems. This article complies with TITAN 2025 guidelines – prohibiting the reporting and use of AI^[5]^.

## A major diagnostic breakthrough: the arrival of the Lumipulse test

In 2025, the FDA approved the Lumipulse G pTau217/β-Amyloid 1-42 Plasma Ratio test, developed by Fujirebio, marking a historic milestone in the biological diagnosis of AD. This test, the first of its kind based on a blood sample, allows for the detection of amyloid markers without the need for PET imaging or a lumbar puncture. It utilizes the Lumipulse platform, which had previously received approval for the β-Amyloid (1-42/1-40) CSF test in 2022^[6]^. The clinical validation trial, conducted with 499 patients, showed an agreement rate of over 90% with reference methods (PET scan or CSF biomarkers). More specifically, 91.7% of patients who tested positive had amyloid pathology confirmed by PET or CSF analysis, while 97.3% of negative results were consistent with the absence of pathology^[7]^. These results demonstrate that the plasma test can rival current diagnostic standards while being simpler, faster, and significantly less expensive (Table 1).

**Table 1:** Summary of two approvals

Approval	Lumipulse G β‑amyloid ratio (1‑42/1‑40)	Lumipulse G pTau217/β‑amyloid 1‑42 plasma ratio
Approval date	May 2022	May 2025
Type of specimen	Cerebrospinal fluid (CSF)	Plasma – dipotassium ethylenediaminetetraacetic acid (K2EDTA)
Method of collection	Lumbar puncture	Routine phlebotomy techniques
Analyte	β-Amyloid 1-42 and β-amyloid 1-40	pTau 217 and β-amyloid 1-42
Diagnostic purpose	Detects amyloid pathology in symptomatic adults	Aids in diagnosis of Alzheimer’s via blood biomarkers
Regulatory pathway	*De novo* authorization + breakthrough device	510(k) clearance
Noninvasive/invasive	Invasive	Noninvasive
Automation/platform	Fully automated via Lumipulse G1200 system	Same Lumipulse platform, adapted for plasma
Assay type	Two-step sandwich immunoassays based on chemiluminescent technology	Same
Accuracy compared to PET/CSF	High agreement with amyloid PET	>90% agreement with PET and CSF biomarker data
Primary clinical setting tested in	Specialized memory clinics	Specialized memory clinics (generalizability pending)
Clinical utility	Improves standardization of CSF testing	First scalable blood-based test for broader access
Use case	Symptomatic individuals with cognitive impairment	Same; not intended for asymptomatic screening

## Democratizing Alzheimer’s diagnosis

This innovation is a game-changer. The Lumipulse makes early, scalable, and accessible screening possible, even outside of specialized centers. In healthcare systems where imaging infrastructure is limited, as in many middle-income countries, it could allow general practitioners to integrate cognitive screening into routine clinical practice. By measuring pTau217 and β-amyloid 1-42 proteins in plasma, the test offers a window into brain pathology even before the onset of clinical symptoms. It thus aligns with the current trend in precision medicine, which focuses on identifying preclinical stages to maximize treatment effectiveness^[1,3,8]^. Its accessibility also opens a new era for translational research, facilitating the recruitment and follow-up of large-scale cohorts. It constitutes a powerful screening tool, allowing for the targeting of patients who require more invasive complementary examinations.

## Methodological promises and limitations

Despite its potential, the Lumipulse test is not a standalone diagnostic tool. Several pre-analytical variables can influence its performance. One study showed that minimal blood contamination (0.25%) or improper sample handling could reduce the measured β-amyloid concentrations^[9]^. Delays in processing or freeze-thaw cycles also affect the stability of the Aβ1-42 and pTau181 markers^[10]^. Furthermore, circadian variations in amyloid and Tau levels can explain up to 50% of the observed variability, although this effect decreases with age^[11]^. The plasma test, while analytically robust, has been validated primarily in specialized memory clinics. Its performance in primary care or among ethnically diverse populations remains to be confirmed^[10,12]^. Finally, plasma biomarkers alone are insufficient to establish a definitive diagnosis. Their interpretation must be based on integrated clinical reasoning, taking into account medical history, neuropsychological test results, and genetic profile, particularly APOE ε4 status^[13]^.

## An innovation that redefines clinical practice

The emergence of tests like Lumipulse is not intended to replace neuroimaging or CSF analysis, but rather to complement the diagnostic process. Used as an initial screening tool, it can reduce the need for costly examinations, improve the detection of early cases, and alleviate the burden on healthcare systems. This two-step diagnostic model, involving a plasma test followed, if necessary, by confirmation with PET or CSF analysis, represents a rational evolution toward more efficient medicine. Beyond its analytical performance, the value of Lumipulse is primarily social and ethical. It democratizes access to diagnosis and reduces inequalities, enabling faster care for patients from disadvantaged backgrounds. It is not just a technological advance; it is a public health revolution.

## Conclusion and perspectives

The FDA approval of Lumipulse G pTau217/β-amyloid 1-42 marks a turning point in the fight against AD. By making diagnosis earlier, less invasive, and more equitable, it brings science closer to the patient and embodies the promise of personalized medicine. But this advance is only a first step. For it to truly transform patient care, it must be accompanied by investments in training, infrastructure, and clinical research. The challenge is not only to diagnose earlier, but to do so better, more humanely, and for everyone.

However, a more accessible diagnosis also raises crucial questions: how do we provide psychological support to patients identified at a preclinical stage? How do we avoid stigmatization and ensure appropriate follow-up care? These issues require a multidisciplinary approach involving neurologists, psychologists, bioethicists, and policymakers. The success of Lumipulse will depend as much on its scientific robustness as on the healthcare system’s ability to integrate it within a humane and ethical framework. Without training for practitioners and a national screening strategy, even the best innovation risks remaining underutilized.

## Data Availability

Not applicable.
